# Using the RE-AIM framework to evaluate the implementation and effectiveness of a WHO HEARTS-based intervention to integrate the management of hypertension into HIV care in Uganda: a process evaluation

**DOI:** 10.1186/s43058-023-00488-2

**Published:** 2023-08-25

**Authors:** Martin Muddu, Fred Collins Semitala, Isaac Derick Kimera, Douglas Joseph Musimbaggo, Mary Mbuliro, Rebecca Ssennyonjo, Simon Peter Kigozi, Rodgers Katwesigye, Florence Ayebare, Christabellah Namugenyi, Frank Mugabe, Gerald Mutungi, Chris T. Longenecker, Anne R. Katahoire, Jeremy I. Schwartz, Isaac Ssinabulya

**Affiliations:** 1 Makerere University Joint AIDS Program (MJAP), P.O. Box 7072, Kampala, Uganda; 2 Makerere University College of Health Sciences, Kampala, Uganda; 3 Infectious Disease Research Collaboration (IDRC), Kampala, Uganda; 4 Ministry of Health, Kampala, Uganda; 5 Department of Global Health, University of Washington, Seattle, WA USA; 6 Section of General Internal Medicine, Yale School of Medicine, 333 Cedar Street, New Haven, CT 06511 USA; 7 Mulago Hospital Complex, Uganda Heart Institute, Kampala, Uganda

**Keywords:** Process evaluation of integrated hypertension-HIV management

## Abstract

**Background:**

World Health Organization (WHO) HEARTS packages are increasingly used to control hypertension. However, their feasibility in persons living with HIV (PLHIV) is unknown. We studied the effectiveness and implementation of a WHO HEARTS intervention to integrate the management of hypertension into HIV care.

**Methods:**

This was a mixed methods study at Uganda’s largest HIV clinic. Components of the adapted WHO HEARTS intervention were lifestyle counseling, free hypertension medications, hypertension treatment protocol, task shifting, and monitoring tools. We determined the effectiveness of the intervention among PLHIV by comparing hypertension and HIV outcomes at baseline and 21 months. The RE-AIM framework was used to evaluate the implementation outcomes of the intervention at 21 months. We conducted four focus group discussions with PLHIV (*n* = 42), in-depth interviews with PLHIV (*n* = 9), healthcare providers (*n* = 15), and Ministry of Health (MoH) policymakers (*n* = 2).

**Results:**

Reach: Among the 15,953 adult PLHIV in the clinic, of whom 3892 (24%) had been diagnosed with hypertension, 1133(29%) initiated integrated hypertension-HIV treatment compared to 39 (1%) at baseline. Among the enrolled patients, the mean age was 51.5 ± 9.7 years and 679 (62.6%) were female. Effectiveness: Among the treated patients, hypertension control improved from 9 to 72% (*p* < 0.001), mean systolic blood pressure (BP) from 153.2 ± 21.4 to 129.2 ± 15.2 mmHg (*p* < 0.001), and mean diastolic BP from 98.5 ± 13.5 to 85.1 ± 9.7 mmHg (*p* < 0.001). Overall, 1087 (95.9%) of patients were retained by month 21. HIV viral suppression remained high, 99.3 to 99.5% (*p* = 0.694). Patients who received integrated hypertension-HIV care felt healthy and saved more money. Adoption: All 48 (100%) healthcare providers in the clinic were trained and adopted the intervention. Training healthcare providers on WHO HEARTS, task shifting, and synchronizing clinic appointments for hypertension and HIV promoted adoption. Implementation: WHO HEARTS intervention was feasible and implemented with fidelity. Maintenance: Leveraging HIV program resources and adopting WHO HEARTS protocols into national guidelines will promote sustainability.

**Conclusions:**

The WHO HEARTS intervention promoted the integration of hypertension management into HIV care in the real-world setting. It was acceptable, feasible, and effective in controlling hypertension and maintaining optimal viral suppression among PLHIV. Integrating this intervention into national guidelines will promote sustainability.

Contributions to the literature

- The increasing burden of hypertension among PLHIV has necessitated the integration of services for hypertension and HIV while leveraging the HIV program gains.
- This is the first real-world implementation study to report on the impact of the WHO HEARTS package on hypertension care among PLHIV.
- Our findings contribute to the literature by reporting implementation and effectiveness outcomes of integrated management of hypertension and HIV using the RE-AIM framework.

## Background

Hypertension is the commonest cause of cardiovascular disease (CVD) among persons living with HIV (PLHIV) [1]. PLHIV with hypertension are at increased risk of stroke, myocardial infarction, and kidney disease [2]. The increasing burden of hypertension among PLHIV has necessitated the integration of services for hypertension and of HIV while leveraging the HIV program infrastructure. Integrated services for hypertension and HIV target to achieve dual control of both diseases while reducing the risk of CVD in the aging HIV population [3].

Strategies to improve hypertension control in the general population have increasingly utilized the World Health Organization (WHO) HEARTS technical packages for cardiovascular control [4]. WHO HEARTS packages for hypertension management include guidelines to promote healthy lifestyle counseling, use of evidence-based treatment protocols, access to medications for hypertension, use of CVD risk-based charts, team-based care for hypertension, and systems for monitoring. The application of the WHO HEARTS guideline to hypertension control in the HIV population has not been studied widely.

To understand the effectiveness of WHO HEARTS packages for hypertension control among PLHIV, we conducted an implementation science project to integrate the management of hypertension and HIV care in Uganda [5]. The study compared both hypertension and HIV outcomes before and after integrating both services using an adapted WHO HEARTS protocol for hypertension management. The key components of this intervention for integrating hypertension services into HIV care included health lifestyle counseling, access to hypertension medications, use of hypertension treatment protocol, task shifting, and systems for monitoring and evaluation. This adapted WHO HEART intervention excluded CVD risk-based charts. We have previously described this adapted WHO HEARTS intervention [5] and reported on its 6-month effectiveness [6].

Here, we conducted a process evaluation through mixed methods to determine the longer-term 21-month effectiveness and key implementation outcomes of the intervention.

The RE-AIM framework with its five domains of reach, effectiveness, adoption, implementation, and maintenance supported the process evaluation. The RE-AIM framework has been widely applied across different implementation strategies including for hypertension. The framework evaluates the robustness of the intervention and factors that affect its generalizability, translation, and sustainability in routine practice [7].

The aim of this study was to determine the long-term effectiveness of the adapted WHO HEARTS-based intervention on hypertension control among PLHIV and evaluate its reach, acceptability, adoption, implementation fidelity, feasibility, and sustainability using the RE-AIM framework.

## Methods

### Study design

This was an explanatory sequential mixed methods study in which we evaluated the effectiveness and implementation outcomes of the WHO HEARTS-based intervention to integrate hypertension and HIV care at a large urban HIV clinic in Uganda. We collected both quantitative and qualitative data concurrently. We then used both quantitative and qualitative methods to complement each other to answer the research questions, using the qualitative to add meaning and perspective to the quantitative findings [8]. We evaluated both the effectiveness and implementation outcomes of integrated hypertension and HIV care after 21 months of implementation using the RE-AIM implementation science framework [9]. RE-AIM has been extensively used and validated across diverse contexts, including clinical settings. We used the RE-AIM framework in the analysis and reporting of both qualitative and quantitative data [10]. Detailed descriptions of the RE-AIM framework, its domains, and its application to this study are provided in Table 1 [7, 9–15]. We followed the Consolidated Criteria for Reporting Qualitative Research (COREQ) and the Standards Reporting for Implementation Studies Checklist (StaRI) in writing the manuscript [16].

**Table 1:** The RE-AIM framework adapted to the evaluation of HEARTS-based HIV/hypertension integration intervention

**RE-AIM dimensions and the operational definitions**	**Indicators/nature of data**	**Measurement and sources of data**
**Reach (mixed methods)**		
Reach is the absolute number, proportion (%), and representativeness of individuals who participated in the integrated hypertension-HIV program.	Number and % of PLHIV who were screened for hypertension compared to baseline Number and % of PLHIV/ hypertension who were started on hypertension treatment compared to baseline Acceptability of integrated hypertension-HIV care	Routinely collected data on hypertension screening from the electronic medical records (EMR) Data on hypertension treatment from the hypertension register and EMR FGDs with PLHIV/hypertension IDIs with healthcare providers and PLHIV/hypertension
**Effectiveness (mixed methods)**		
Effectiveness is the impact of integrated hypertension-HIV care on outcomes, including potential negative effects, quality of life, and economic outcomes.	Number and % PLHIV/hypertension retained in hypertension/HIV treatment at 21 months Number and % PLHIV/hypertension with controlled hypertension at 21 months compared to baseline Number and % PLHIV/hypertension with HIV control (VL < 1000) at 21 months compared to baseline Number and % PLHIV/hypertension with side effects of hypertension treatment Number and %PLHIV/hypertension LTFU, transferred, and dead Side effects, quality of life, cost savings, and healthy lifestyle	Data on hypertension treatment from the hypertension register and EMR Data on viral load monitoring from the EMR IDI with healthcare providers and PLHIV/hypertension KII with healthcare providers and PLHIV/hypertension
**Adoption (mixed methods)**		
Adoption is the absolute number, proportion, and representativeness of healthcare providers, HIV clinic leaders, and MoH leaders who are willing to initiate and incorporate integrated hypertension-HIV care into HIV clinical settings.	Number and % of healthcare providers trained on WHO HEARTS protocol Number and % healthcare providers who were willing to participate in delivering integrated hypertension-HIV care compared to baseline Healthcare providers’ perception about the comfort, appropriateness, and relative advantage of integrated hypertension-HIV care	Attendance lists of healthcare provider training Review of patient screening and treatment records in EMR KII with healthcare providers at Mulago ISS Clinic
**Implementation (mixed methods)**		
Implementation refers to the healthcare provider’s and supervisors’ fidelity to the various elements of integrated hypertension-HIV care	Number and % of all planned activities implemented (training healthcare providers, SOPs, availability of tools for hypertension care) Barriers and facilitators to implementation	Review of the activity log for the project Review of patient screening and treatment records in EMR IDIs with the healthcare providers KII with MoH policymakers
**Maintenance (mixed methods)**		
Maintenance is the extent to which integrated hypertension-HIV care became institutionalized or part of the routine HIV clinical practices and policies as well as sustainment over time and reinforcing factors.	Active hypertension/HIV focal person peers *N* (%) follow-up contacts for clients in hypertension/HIV care Leveraging HIV program resources for integrated hypertension-HIV care Hypertension care integrated and supported in the national HIV program Number and adequacy of job aides available for use Availability of hypertension medicines and BP machines	Review of patient screening and treatment records in EMR Physical counting of BP machines IDIs with healthcare providers and PLHIV/hypertension KIIs with clinic leaders and MoH policymakers

*FGD* focus group discussion, *KII* key informant interview, *IDI* in-depth interview, *LTFU* loss to follow-up, *M&E* monitoring and evaluation, *EMR* electronic medical records

### Study setting

We conducted the study at Mulago immune suppression syndrome (ISS), a large HIV clinic in Kampala, Uganda, that provides comprehensive HIV services to over 16,500 PLHIV. The clinic is located within the Mulago National Referral and Teaching Hospital Complex and is owned and operated by the Makerere University Joint AIDS Program (MJAP). HIV clinical services at Mulago ISS include HIV testing, counseling, and provision of ART. All available medicines and services at the clinic are provided to patients at no cost.

In line with the Uganda National Guidelines for HIV care [17, 18], PLHIV are routinely screened for hypertension. Prior to the study commencement, the clinic had already achieved universal screening for hypertension among all PLHIV during each clinic visit and had surpassed the 95% target of initiating ART and viral suppression among PLHIV. The blood pressure for all patients was measured in a sitting position after 5 min of rest. Blood pressures initially recorded as > 140/90 mmHg were measured again twice within 2 min, for a total of three measures. The lowest reading of the three was the final record. The same automated blood pressure machine (Omron-M6-HEM-7321-E) was used to measure blood pressure both at baseline and on every follow-up visit. We reviewed the previous blood pressure recordings for patients with persistently elevated blood pressures in the electronic medical records at least a month earlier before diagnosing hypertension and recommending treatment. The expert clients screened PLHIV for hypertension while confirmatory blood pressure measurements were taken by a clinic nurse. Both the nurse and the expert clients were trained on standardized blood pressure measurements at the commencement of the study.

This study was funded by Resolve to Save Lives, through its Learning, Implementation, Networking, Knowledge, and Support (LINKS) program.

### Components of the WHO HEARTS-based intervention for integrated hypertension-HIV care

**Fig. 1:**
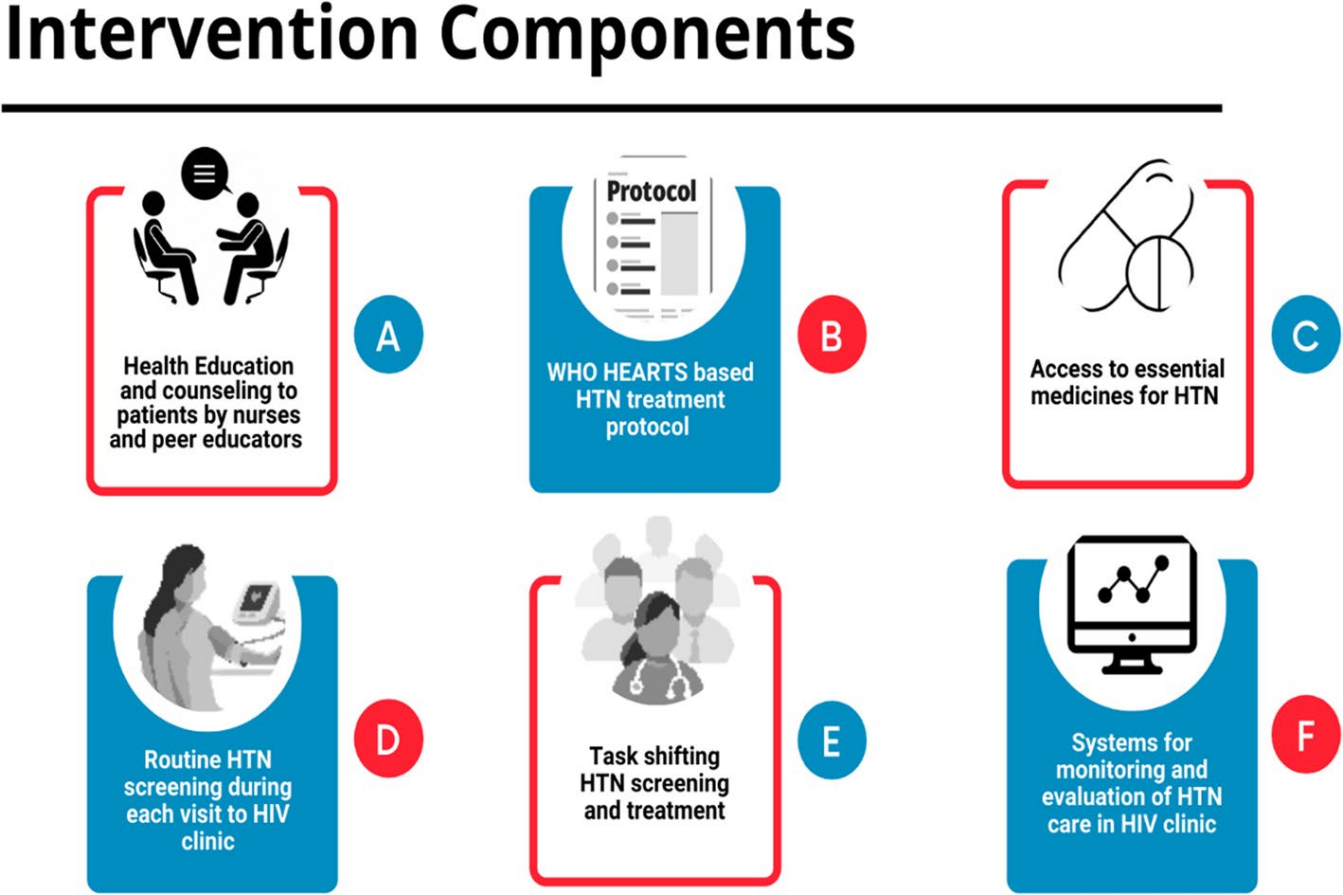
Components of the HEARTS-based intervention for integrated management of HIV and hypertension. **A** Ccounseling and support on adherence to both hypertension and HIV medicines, side effects of medicines, implementation of physical exercise, healthy diet, salt reduction, weight reduction, and smoking cessation. **B** Simple, stepwise approach to titrate amlodipine, valsartan, and hydrochlorothiazide as the first-, second-, and third-line therapies, respectively. **C** Procured amlodipine, valsartan, and hydrochlorothiazide from a private not-for-profit access program and we gave medicines to patients at no cost. **D** Blood pressure (BP) measured by a peer educator using a validated Omron M6 BP machine. Clinicians repeated the BP measurement for patients with initial BP > 140/90 mmHg. **E** In addition to the aforementioned task shifting of measuring BP, we trained and mentored clinical officers and nurses to prescribe hypertension medications to reduce the burden on doctors. **F** Developed and shared quarterly targets on hypertension care indicators with healthcare providers. We adapted the HEARTS hypertension register and CVD patient cards and utilized them to record patient data. Conducted quarterly performance review meetings

The six components of the HEARTS-based intervention for integrated HIV-hypertension care that we implemented have been previously described [6] and are summarized in Fig. 1.

### Implementation strategies

*Training of healthcare providers*: The research team trained healthcare providers on the use of the adapted WHO HEARTS intervention in eight 2-h sessions. *Integrated clinic visits*: For PLHIV diagnosed with hypertension, treatment for both HIV and hypertension was offered at the same clinic by the same healthcare provider during the same clinic visit. *Patient identifiers*: To facilitate the identification of hypertension patients, healthcare providers attached unique stickers on files of PLHIV diagnosed with hypertension. *Synchronized appointments*: The attending health worker synchronized the follow-up appointment dates to review both hypertension and HIV concerns. *Technical supervision*: The research team and two technical officers from the Department of NCDs at the Uganda Ministry of Health provided quarterly technical supervision and feedback to healthcare providers to adhere to the study activities.

## Quantitative methods

### Study population

We included all PLHIV aged ≥ 18 years diagnosed with hypertension at Mulago ISS Clinic from August 1, 2019, the start of our project, to April 30, 2021, the end of the project.

### Data collection

For the quantitative component of the study, we extracted routinely collected patient data on hypertension and HIV care from the electronic medical records (EMR). We collected data on the number of PLHIV screened for hypertension and the number of patients diagnosed with hypertension who enrolled in integrated hypertension-HIV care. We determined monthly proportions of hypertensive PLHIV with controlled hypertension (BP < 140/90 mmHg) and controlled HIV (viral load < 1000 copies/ml). We measured the above hypertension and HIV outcomes in a cohort of patients enrolled on integrated HIV-hypertension at baseline and monthly up to 21 months of implementation.

### Data analysis

We conducted univariate analyses to describe the baseline characteristics of adult hypertensive PLHIV who received integrated HIV-hypertension care at the clinic. We obtained the means and standard deviations for continuous variables and percentages and frequencies for categorical variables. We then categorized the data into two sub-groups: those that enrolled in integrated hypertension-HIV care and those that did not. We then compared the baseline characteristics of these two subpopulations using the chi-square or Fisher’s exact tests for categorical variables and *t*-tests for continuous characteristics.

To investigate the effectiveness of the intervention, quantitative data analysis included descriptive analyses using monthly frequencies and proportions to evaluate trends of % hypertension and % HIV control at baseline and monthly after introducing the intervention. In addition, we measured the % retention at 21 months, % mortality, % loss to follow-up, and % occurrence of side effects for hypertension medicines. We evaluated the effectiveness of the intervention by comparing the treatment outcomes at baseline before introducing the intervention and after 21 months of implementation using the paired *t*-test to account for within-subject variation. We analyzed data using STATA version 16.

## Qualitative methods

### Study population

We purposively selected hypertensive PLHIV as a sub-set of participants in the quantitative study and healthcare providers caring for PLHIV for the interviews. Hypertensive PLHIV were eligible if they had been receiving integrated hypertension-HIV for at least 1 year and did not have a cognitive impairment that precluded their active participation in the interviews. We approached patients through telephone calls close to their next scheduled clinic appointment to introduce the study and inquire about their willingness to participate in the interviews. Eligible healthcare providers were individuals of different health professions who had been providing care to patients at the Mulago ISS Clinic for at least 6 months. We approached eligible healthcare face to face.

Furthermore, we selected policymakers from the Uganda Ministry of Health non-communicable disease (NCDs) department who had supervised the implementation of integrated HIV-hypertension care at Mulago ISS Clinic for at least 1 year. The selected policymakers had supervised similar projects on integrating NCDs management into HIV care.

### Data collection

We used semi-structured interview (SSI) guides that were developed based on the research questions. All interviews had open-ended questions to explore patients’ and healthcare providers’ perspectives regarding the implementation of integrated HIV-hypertension care using the WHO HEARTS-based intervention. Prior to data collection, we pretested the interview guides with hypertensive PLHIV and healthcare providers at the Mulago ISS Clinic who were not participating in the study. FA, RS, and MM shared the objectives of the evaluation phase of the study with clinic leaders, healthcare providers, and patients who had implemented integrated hypertension services in the last 21 months since the start of the intervention. Hypertensive PLHIV who had received integrated hypertension services at Mulago ISS Clinic within the 21-month intervention period were invited to participate in focus group discussions (FGDs) and semi-structured in-depth interviews (IDIs) to explore both the group and individual lived experiences and perceptions regarding integrated hypertension-HIV care. All the interviewers were trained social scientists with expertise in qualitative research including conducting FGDs and semi-structured interviews. The interviewers established a rapport with the clinic leaders, healthcare providers, and patients prior to study commencement, but were not part of the healthcare team at the clinic, thus limiting potential bias. We conducted four FGDs with each group consisting of six patient participants. Each session lasted approximately 60 min. FGDs explored participants’ experiences of receiving integrated hypertension care during the past 21 months of the intervention. We sought to explore the changes in BP control, changes in lifestyle, socio-economic conditions, and other factors that may have been impacted by the intervention. We conducted six semi-structured IDIs with hypertensive PLHIV each lasting 30–45 min.

We conducted eight (8) key informant interviews (KIIs) with healthcare providers at the clinic. The KIIs explored healthcare providers’ experience with providing hypertension care in the HIV clinic, existing gaps in hypertension care, training needs for staff, and recommendations for improvement. The healthcare providers comprised doctors, nurses, clinical officers, pharmacy technicians, and hypertension-HIV peer educators. These were purposely selected to represent the various healthcare professionals in the health facility. In addition, we interviewed two policymakers from Uganda’s MoH Department of non-communicable diseases who closely provided technical supervision during the implementation period. The interviews explored the views regarding the implementation of integrated HIV-hypertension care at the HIV clinic and the potential of sustainable scaling up across the country. We recruited interview participants until data saturation. Data saturation was realized when no new information emerged from the interviews and focus group discussions. All KIIs were conducted in English while IDIs and FGDs were conducted in Luganda the local language in the area of study. All interviews were audio-recorded and transcribed verbatim in Luganda and then translated into English.

### Data analysis

A research team with expertise in public health, social science, and clinical care (MM, FA, and RS) conducted the thematic content analysis. The team coded transcripts using an inductive approach. The coding process was guided by consensual qualitative research (CQR) procedures [19]. First, each team member read four transcripts independently and identified preliminary codes. Through a series of meetings, the team agreed on an initial set of codes. All transcripts were coded and analyzed in the Atlas.ti (version 8) software. FA checked all transcripts for accuracy and completeness before they were uploaded into the ATLAS.ti V8 software. IK independently coded eight of the transcripts to establish inter-coder reliability (kappa 0.80). Through subsequent meetings, researchers developed the final codebook. The codes were then categorized into sub-themes, and these were mapped onto the RE-AIM domains and constructs. Code reports were generated centrally. FA synthesized the findings and summarized them into themes based on the domains of the RE-AIM framework. We extracted illustrative quotations from the transcripts.

We maintained privacy during data collection, analysis, and storage. All identifiable information was removed from final records after data collection to ensure participant anonymity, and only the core research team and the principal investigator had access to the original data. Access to data for statistical analysis by other individuals was limited to de-identified data.

### Data validation and feedback to study participants

We conducted a learning session with all healthcare providers in the clinic and shared the results of this study and received feedback. In addition, we disseminated the findings of this study to patients during the routine health education talks at the clinic. The healthcare providers and patients at the clinic expressed that the results were representative of their perceptions and experiences regarding integrated HIV-hypertension care. We also disseminated these findings to the technical working group of the Uganda MoH that is working to integrate the management of hypertension and other NCDs into HIV care.

## Results

We present the results according to the RE-AIM framework below.

### Reach

Among the 15,953 adult PLHIV in the clinic, of whom 3892 (24%) had been diagnosed with hypertension, 1133 (29%) agreed to participate in integrated HIV and hypertension care. Universal screening for hypertension had been achieved prior to study commencement and was sustained throughout the study. At 21 months, all 1133 (100%) PLHIV diagnosed with hypertension, who had agreed to participate in integrated HIV-hypertension care and had made at least one clinic visit, were initiated on hypertension treatment according to the treatment protocol compared to 39 (1%) at baseline.

Of the enrolled patients, 679 (62.6%) were female. The mean age was 49.5 ± 9.7 and 52.1 ± 9.5 years for females and males, respectively. A total of 282 (26%) participants were obese (body mass index > 30 kg/m^2^), 512 (47.2%) had a baseline CD4 count of less than 200 cells per mm^3^ at ART initiation, and 887 (81.8%) had been on ART for 5 years or more (Table 2).

**Table 2:** Characteristics of hypertensive PLHIV at baseline (*N* = 3892)

**Characteristic**	**Patients not enrolled in the cohort (*n* = 2759)**	**Patients enrolled in the cohort (*n* = 1133)**	***P*-value**
Age in years, mean (SD)	44.7 (10.2)	50.2 (9.6)	< 0.001
Age categories, count (%)			
18–29	157 (5.7)	15 (1.3)	0.001
30–39	753 (27.3)	144 (12.7)	
40–49	1013 (36.7)	400 (35.3)	
50 and older	836 (30.3)	574 (50.7)	
Gender, count (%)			
Male	1084 (39.3)	427 (37.7)	0.210
Female	1675 (60.7)	706 (62.3)	
Baseline BP in mmHg, mean (SD)			
Systolic	143.0 (15.2)	154.2 (20.8)	< 0.001
Diastolic	92.6 (9.3)	97.8 (13.3)	< 0.001
Baseline BMI, count (%) (*n* = 3730)			
Underweight (< 19.0)	254 (9.2)	62 (5.5)	< 0.001
Normal weight (19.0 to < 25.0)	1258 (45.6)	392 (34.6)	
Overweight (25.0 to < 30.0)	739 (26.8)	376 (33.2)	
Obese (> 30.0)	508 (18.4)	303 (26.7)	
Baseline CD4 category, count (%)			
< 50	353 (12.8)	127 (11.2)	< 0.001
50 to < 100	207 (7.5)	70 (6.2)	
100 to < 200	359 (13.0)	213 (18.8)	
≥ 200	1840 (66.7)	723 (63.8)	
Baseline ART regimen, count (%) (*n* = 3878)			
AZT-3TC-NVP	930 (33.7)	480 (42.4)	< 0.001
AZT-3TC-EFV	331 (12.0)	211 (18.6)	
TDF-3TC-NVP	212 (7.7)	110 (9.7)	
TDF-3TC-EFV	974 (35.3)	268 (23.7)	
Others	312 (11.3)	64 (5.6)	
ART duration (years)			
< 2 years	568 (20.6%)	51 (4.5%)	< 0.001
2–5 years	748 (27.1%)	239 (21.1%)	
5–10 years	1179 (42.7%)	539 (47.6%)	
> 10 years	264 (9.6%)	304 (26.8%)	

*BMI* body mass index, *ART* antiretroviral therapy, *BP* blood pressure, *TDF* tenofovir, *3TC* lamivudine, *DTG* dolutegravir, *ABC* abacavir, *EFV* efavirenz

Characteristics of PLHIV diagnosed with hypertension who participated in the qualitative interviews (*n* = 30), healthcare providers (*n* = 9), and policymakers (*n* = 2) are summarized in Table 3. The majority of participants (27 (65.9%)) were female.

**Table 3:** Number and characteristics of participants for the qualitative study

**Data collection methods**	**Number and category of participants**	**Total participants**	**Female, *N* (%)**	**Mean age (SD)**
Focus group discussions (FGDs) for patients	Patients who had both hypertension and HIV (4 FGDs)	24	18 (75.0%)	53.5 (± 7.2)
In-depth interviews (IDIs) for patients	Patients who had both hypertension and HIV (6 IDIs)	6	3 (50.0%)	55.3 (± 7.7)
Key informant interviews (KIIs) for healthcare providers	Doctor	3	2 (66.7%)	N/A
	Nurse	2	1 (50.0%)	
	Clinical officer	1	1 (100.0%)	
	Pharmacy technician	2	1 (50.0%)	
	HIV/hypertension peer educator	1	1 (100%)	
	MoH policymakers	2	0(0.0%)	
**Total number of participants**		**41**	**27 (65.9%)**	

According to PLHIV with hypertension, integrated HIV-hypertension care was more acceptable compared to separate services. Patients were happy and comfortable to receive integrated services for hypertension and HIV in one clinic compared to separate services:

*“The treatment of hypertension helped us to reduce the costs and the time we could take moving from one place to another accessing treatment. It saved us from attending the HIV clinic on one day and then a hypertension clinic the following week.” (FGD 004, Participant 5).*

Furthermore, healthcare providers reported that integrated hypertension-HIV care benefited patients as it enabled them to save time, costs of transport, and medication:

*“They receive all the care at the same place which saves their time. They reduce transport costs and do not have to purchase those medications.” (KII, Health Care Provider, 005).*

### Effectiveness

Effectiveness related to the impact of the intervention on both hypertension and HIV outcomes. There was a significant improvement in hypertension control from 9% at baseline to 72% at month 21 among PLHIV enrolled in integrated HIV and hypertension care (*p* < 0.00l). Healthcare providers noted that: “Registering hypertension control of up to 72% among patients treated for hypertension is infrequent in resource limited settings;

*we were used to seeing blood pressure control of around 10%. We can leverage the HIV structure for improved hypertension control.” (KII, Policy maker, 001).*

**Fig. 2:**
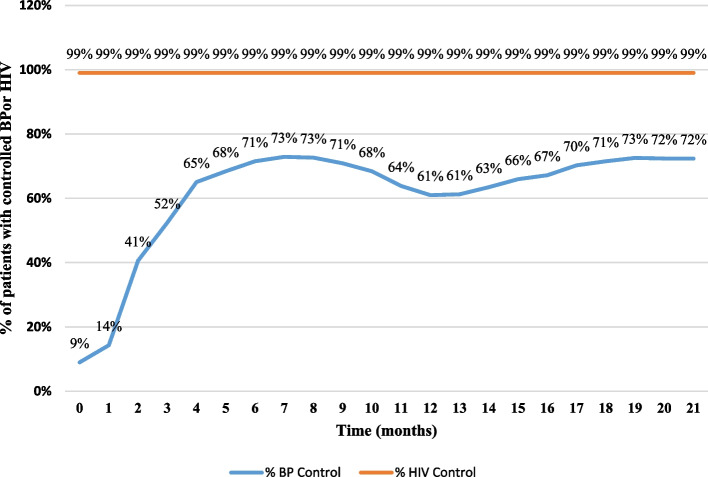
Percentage of patients enrolled in integrated hypertension-HIV care with controlled BP and suppressed HIV viral load (*N* = 1133). This is longitudinal patient-level data

Overall, the monthly trend of hypertension control among PLHIV with hypertension receiving integrated HIV-hypertension care was positive until March 2020 when COVID-19 emerged in Uganda. At the beginning of the COVID-19 pandemic, the monthly hypertension control declined until September 2020. Hypertension control then improved until April 2021 (Fig. 2).

There was improved adherence to a healthy lifestyle by the patients who received integrated HIV-hypertension care which could have contributed to hypertension control as well:

*“I eat greens. I have minimized sugary foods. I avoid foods with a lot of oil and regulate the amount of salt I eat and avoid alcohol. I jog for at least 30 minutes other than sitting.” (FGD Participant, 002).*

The mean systolic blood pressure improved from 153.2 ± 21.4 mmHg at baseline to 129.2 ± 15.2 mmHg at 21 months (*p* < 0.001). Likewise, the mean diastolic blood pressure improved from 98.5 ± 13.5 mmHg at baseline to 85.1 ± 9.7 mmHg at 21 months (*p* < 0.001) (Table 4).

**Table 4:** Comparison of hypertension and HIV outcomes at baseline and 21 months among patients enrolled in integrated hypertension/HIV care

**Outcome variable**	**Baseline (*N* = 1133)**	**21 months (*N* = 1133)**	***P*-value**
Mean systolic BP, mmHg (± SD)	153.2 ± 21.4	129.2 ± 15.2	< 0.001
Mean diastolic BP, mmHg (± SD)	98.5 ± 13.5	85.1 ± 9.7	< 0.001
Patients with controlled hypertension, *N* (%)	102 (9.0)	820 (72.4)	< 0.001
Patients with controlled HIV (%)	1125 (99.3)	1127 (99.5)	0.694

*SD* standard deviation, *BP* blood pressure

Patients who received integrated HIV-hypertension care reported improved quality of life that could have resulted from reduced blood pressure:

*“Since we started getting the hypertension medicine from the clinic, my blood pressure levels have stabilized. I am feeling healthy, strong and I do not fall sick often. I do my work.” (In-depth Interview with patient 001).*

Additionally, patients who received integrated hypertension-HIV care noticed increased savings on out-of-pocket hypertension medicine expenditure: 

*“Getting hypertension medicines helped to minimize the expenditures on hypertension medications. I have saved the money for other needs like school fees and feeding my family.” (FGD 001, Participant R1).*

Despite integrating hypertension services into HIV care, the serum HIV viral load control among hypertensive PLHIV did not change significantly, ranging from 99.3% at baseline to 99.5% at 21 months (*p* = 0.694) (Fig. 2).

Overall, 1087 (95.9%) of all the patients enrolled in integrated hypertension-HIV care were retained throughout the 21 months. Of the 46 (4%) participants who were not retained, 15 (1.3%) were transferred to HIV clinics nearer their homes on request, and 25 (2.2 %) were lost to follow-up. Six (0.5%) patients died of liver cancer (*n* = 1), kidney failure (*n* = 2), heart attack (*n* = 1), and stroke (*n* = 2). Only 10 (0.9%) patients experienced side effects from amlodipine including lower limb edema (*n* = 5) and headache (*n* = 5) necessitating the substitution of their hypertension medication. Healthcare providers noted that the quality of hypertension medications was good and helped patients to achieve hypertension control with few side effects:

*“The side effects are less compared to other medications patients were taking previously. With the integration, patients had hypertension controlled, which was not the case previously.” (KII, Health Care Provider, 005).*

Additionally, patients who received integrated hypertension-HIV care reported relief of worries associated with the cost of buying medicines for hypertension:

*“I used to be worried if I would afford hypertension medicines. However, since they started giving us hypertension medicine, I feel relieved.” (FGD, 002 Participant R4).*

### Adoption

#### Task shifting in measuring blood pressure and prescribing hypertension medications facilitated integrating hypertension and HIV care

This approach allowed healthcare providers that were not previously involved in screening and treating hypertension to do so. PLHIV peers were supported to screen for hypertension while nurses and non-physician healthcare providers (clinicians with diploma-level training) were trained to prescribe hypertension medications.

*We have had a lot of engagement with PLHIV peers given that patients open up to them more easily. Shifting hypertension screening to peers reduced the workload of some health workers. (KII, Health Care Provider, 007).*

A total of 18 (86%) health workers including doctors, clinical officers, and nurses were empowered and prescribed hypertension medications within 21 months of project implementation compared to 5 (24%) clinicians at baseline. At baseline, only doctors prescribed hypertension medications.

*Before the LINKS project, only doctors were prescribing hypertension medications. Any patient with hypertension would be referred to a doctor within the clinic. However, the project built our capacity and gave us guidelines that doctors, non-physician healthcare providers, and nurses can utilize to prescribe hypertension medications and consult when uncomfortable. (KII, Health Care Provider, 008).*

#### The trained healthcare workers provided integrated hypertension-HIV care

A total of 48 (96%) healthcare providers in the clinic were trained on the implementation of the WHO HEARTS protocol, reflecting providers’ interest in integrated HIV-hypertension care. This enabled task shifting, which was not previously the case.

*As a nurse, I was empowered to prescribe hypertension medications to stable patients by using a treatment protocol. The counsellors are able to provide adherence support for both hypertension and ART medications. (KII, Health Care Provider, 004).*

### Implementation

We conducted 10 (125%) of the planned eight training sessions on integrated HIV-hypertension care for healthcare providers. Incorporating the trainings into the clinic’s weekly continuous medical education sessions enabled us to deliver two additional trainings. The hypertension treatment protocols and standard operating procedures (SOPs) for integrated HIV and hypertension were printed and distributed to all 12 (100%) clinical rooms following the training on their use. The MoH conducted 6 (75%) of the planned eight technical supervision visits to the clinic during the study period. The clinic pharmacist utilized the MoH-recommended electronic logistics information systems for medicine management to forecast, quantify, procure, and monitor stock levels for both ART and hypertension medicines. The clinic did not experience stock out of hypertension medicines during the study period.

#### Fidelity of implementing integrated hypertension-HIV care

Integrated HIV-hypertension care was consistently implemented as planned. Healthcare providers received tools to guide the implementation and were trained to use them.

*I was trained on the management of hypertension and HIV and received the necessary tools. I was educated on using the hypertension treatment protocols, standard operating procedures and filling the patients’ charts. The patients’ charts have stickers so it is easy to identify patients diagnosed with HIV and hypertension. (KII, Health Care Provider, 005).*

#### Training healthcare providers facilitated fidelity of integrated hypertension-HIV care

At the start of the project, we trained healthcare providers on hypertension care and treatment. Staff teamwork, efficient flow of patients at the clinic, and proper record-keeping facilitated fidelity.

*Following the training, we have embraced teamwork in managing PLHIV with hypertension. The HIV clinic flow and the recordkeeping are favourable; I feel that I have been well supported. (KII, Health Care Provider, 008).*

#### Task shifting facilitated fidelity of implementation

Policymakers mentioned that task shifting facilitated the seamless implementation of integrated and holistic hypertension-HIV care and recommended its scale-up.

*Being able to provide holistic care has given a rewarding experience to healthcare providers here. We have demonstrated task shifting through expert clients who measure blood pressure with the supervision of qualified staff. Nurses participate in prescribing ART and hypertension medications. That is exactly what we would want as the government in view of our human resource challenges. (KII, policy maker, 002).*

#### Feasibility of integrated hypertension-HIV care

Healthcare providers implemented integrated hypertension-HIV care seamlessly with the available resources in the HIV clinic.

*We screen for hypertension in all adult PLHIV because we have been trained and have adequate blood pressure machines. Regarding hypertension medication, we have the calcium channel blocker, amlodipine and angiotensin receptor blocker, valsartan. (KII, Health Care Provider, 005).*

#### Synchronized hypertension and HIV clinic appointments strengthened the integration

Healthcare providers synchronized days of refill for hypertension medications and ART among all 1133 hypertensive PLHIV who received integrated hypertension-HIV care.

*When patients come for their ART, ‘we provide them with their hypertension medication refill as well.’ (KII, Health Care Provider, 006).*

Additionally, MoH policymakers reflected on the providers’ experience with integrated hypertension-HIV services:

*“Every provider wants to have a healthy client; so if all the client’s ailments are controlled then the provider is happy.” (KII, Policy Maker, 002).*

#### Routinized screening of all PLHIV for hypertension during each clinic visit promoted the integration of hypertension and HIV services

PLHIV with hypertension were managed for both conditions in an integrated fashion. Healthcare providers innovated file identifiers and data tools for all 1133 PLHIV with hypertension:

*“We take blood pressure for all our patients at every visit. If the blood pressure is high and the patient needs treatment, we give them hypertension medications and monitor them. On every file of hypertension patients, we put a unique sticker so healthcare providers can easily identify them.” (KII, Health Care Provider, 008).*

#### The relative advantage of integrated hypertension-HIV compared to separate services

Healthcare providers preferred implementing integrated hypertension-HIV care to separate services.

*Integration is the way to go because it gives the patient a comprehensive package. (KII, Health Care Provider, 004).*

Healthcare providers at the clinic granted a referral to PLHIV with hypertension, upon request, to access treatment for hypertension from other health facilities. However, other adaptations to program components were not studied qualitatively.

### Maintenance

#### Leveraging HIV program resources to integrate and sustain the management of hypertension among PLHIV

Policymakers at the MoH believed that integrating the management of hypertension and HIV was sustainable. Leveraging the HIV infrastructure and resources to address the chronic care needs of PLHIV with multiple comorbidities like hypertension and diabetes mellitus would foster sustainability.

*We have learned that chronic conditions can be managed together without necessarily adding resources. We will write policy briefs to guide us on how to take up this kind of integration. We will not integrate hypertension alone; we will include diabetes mellitus. (KII, Policy maker, 001).*

#### The hypertension treatment protocols would foster the sustainability of integrated hypertension-HIV care at the clinic

Healthcare providers reported that it would be possible to sustain integrated hypertension-HIV care since they had the hypertension treatment protocols.

*Every clinical room has a simplified step-wise hypertension treatment protocol, which guides providers on which medicines they should give at different stages of hypertension. (KII, Health Care Provider, 003).*

#### MoH was interested in adopting a national hypertension treatment protocol for national scale-up

Policymakers at the MoH got interested in scaling up the integrated hypertension-HIV program including adopting a national hypertension treatment protocol.

*We plan to adopt and scale up nationally a similar single-page, stepwise treatment protocol as the one used by Makerere University Joint AIDS Program (MJAP). With this evidence, we shall revise our policies and treatment guidelines. (KII, Policy maker, 001)*

#### MoH recommended revision of the National Essential Medicines List

Policymakers at the Ministry of Health noted that the lessons learned from the integrated hypertension-HIV project at MJAP will inform the revision of the national essential medicines list to include newer and low-cost hypertension medicines as used on the MJAP hypertension treatment protocol. To improve hypertension control nationally, a regular supply of hypertension medicines would be needed.

*We will include newer, low-cost and effective hypertension medicines in our essential medicines list to be available to patients nationally. With regular supply of medicines, we can achieve optimal hypertension treatment control. (KII, Policy Maker, 002)*

## Discussion

In this evaluation study, we report a feasible, effective, acceptable, and sustainable WHO HEARTS-based intervention for integrated HIV-hypertension care at a large urban HIV clinic in Uganda.

A third of all PLHIV with hypertension were initiated on treatment for hypertension. The relatively low reach of hypertension treatment among hypertensive PLHIV was attributed to some patients who preferred hypertension treatment regimens other than our protocol medication. Additionally, PLHIV with hypertension who requested a referral to other health facilities to access treatment for hypertension were granted a referral.

There was high acceptability of the WHO HEARTS-based intervention as patients, providers, and policymakers preferred integrated hypertension-HIV care to vertical or separate services. This was attributed to the improved convenience and client-centeredness of the integrated services. In addition, patients added that receiving hypertension services at the HIV clinics at no cost was preferable since most hypertension medicines were unaffordable to most patients, thus affirming the acceptability of integrated services.

Receiving hypertension medicines at no cost enabled patients to minimize out-of-pocket expenditures for treating hypertension. Similarly, in a scoping review, PLHIV receiving integrated hypertension-HIV care reported reduced transport costs and time away from work compared to separate services [20]. Also, in Tanzania, PLHIV with NCD comorbidity expressed a preference for integrated HIV and NCD services due to early identification and management of comorbidities [21].

The integrated HIV and hypertension strategy was effective in improving the proportion of hypertensive PLHIV with controlled hypertension from 9 to 72% in 21 months. The improvements in hypertension control were associated with improvement in quality of life and symptom relief as reported by PLHIV. Lee et al. observed that patients with controlled hypertension reported better quality of life and were at lower risk of cardiovascular-related morbidity and mortality [22].

While integrated HIV and hypertension care has been piloted earlier in SSA, no study has registered such high levels of improvement in hypertension control [3, 23]. This may be attributed to the differences in the interventional components employed by the different studies and differences in the denominator population, that is, all patients with hypertension versus those who agreed to participate in a program. Most of the prior studies reported limited access to a free and consistent supply of hypertension medicines to patients, which directly impacted the control step of the cascade. Elsewhere in North California, higher hypertension control was attained in a community hypertension control program that instituted hypertension management components similar to the WHO HEARTS package [24].

Our gains in hypertension control were threatened by the COVID-19 pandemic and related restrictions to movement in Uganda. In March to June 2020, Uganda had a national lockdown with restrictions on movement. Many patients would not access their primary HIV clinics. Those that managed to get ART from nearby HIV clinics were not able to receive hypertension treatment leading to a drop in hypertension control levels. The trend of improvement was regained after lifting the national lockdown. Overall, NCD services were negatively impacted during the COVID-19 pandemic, underscoring a need for integrated services [25].

There was no significant difference in the HIV viral load control rates from baseline (99.3%) to the 21 months’ mark (99.5%), highlighting that integrating the management of hypertension into HIV care does not necessarily disrupt HIV care. On the other hand, integrating HIV and hypertension care within the HIV clinics would preserve the HIV program gains by reducing the risk of adverse hypertension-related morbidity and mortality in the aging population of PLHIV. The level of retention of PLHIV with hypertension receiving integrated care was optimal at 97%, higher than the retention in the general HIV clinic population of 92%. A similar HIV and hypertension study in Haiti reported optimal retention in integrated care at 82% [26].

Integrated care promotes task shifting and task sharing. Before integrating HIV and hypertension care at the clinic, only doctors prescribed hypertension medications. Following trainings in integrated care, nurses and non-physician healthcare providers (clinicians with diploma-level training) prescribed hypertension medications guided by the treatment protocol. This interprofessional approach to hypertension management lessens the workload on doctors who concentrate on very sick patients.

Simplified hypertension treatment protocols have been used earlier to simplify hypertension management in the general population and are promising to facilitate integrated HIV-hypertension care in low-resource countries [27]. Embracing task sharing, a successful HIV program best practice for hypertension management will facilitate HIV and hypertension integration, especially in high-volume HIV clinics.

Furthermore, hypertension screening was initially done by nurses, but later PLHIV peers were trained to screen for hypertension at triage using automated BP machines. Task shifting of hypertension screening to expert clients enabled nurses to support more skilled roles at the clinic and to prescribe for hypertension. Across sub-Saharan Africa, PLHIV peers have participated in HIV care and are a good resource in hypertension care [28].

Earlier studies in Sub-Saharan Africa recommended a need to leverage the resources of the HIV program and adopt best practices in HIV care to improve hypertension control among PLHIV [23]. Stakeholders noted that sustaining integrated HIV and hypertension care is feasible while leveraging the HIV program resources. In most low- and middle-income countries, the HIV program has been better resourced compared to hypertension services. Training the health providers and availability of simplified protocols for screening and treatment of hypertension among PLHIV would further facilitate the continued provision of integrated HIV and hypertension care. Studies that have evaluated the implementation of the WHO HEARTS package have been in South America. These studies have demonstrated the feasibility and adoption of the HEARTS package but did not report on hypertension control outcomes [29, 30].

## Conclusions

This study illustrated the reach, acceptability, effectiveness, adoption, fidelity, feasibility, and sustainability of integrated HIV and hypertension care based on the adapted WHO HEARTS intervention. This is the first implementation study to report on the impact of the WHO HEARTS package on hypertension care among PLHIV in the real-world setting. Successful scale-up of integrated hypertension-HIV care will necessitate benchmarking on HIV program best practices, adopting simplified national hypertension treatment protocols, and improving access to hypertension medicines at HIV clinics.

### Limitations of the study

We conducted the study at a successful urban HIV clinic meeting most of the UNAIDS targets of identifying at least 95% PLHIV, treating at least 95% of those identified, and achieving viral suppression in at least 95% of PLHIV on treatment. The findings of this study are generalizable to similar HIV clinics. This clinic may not be representative of HIV clinics that are trailing on the UNAIDS 95-95-95 targets.

We did not collect and analyze data from patients who declined enrollment in the study. Thus, we were unable to explore patient-level factors for non-participation as well as undesirable aspects of the intervention. However, this is a priority for future research.

There were significant differences in some aspects like baseline mean blood pressure between the individuals that accepted to enroll for integrated HIV and hypertension care versus those that declined. These could have influenced hypertension outcomes during the follow-up period. It would have been beneficial to determine outcomes among participants that declined integrated care.

Additionally, we were not able to collect data on the number and proportion of each type of healthcare providers that prescribed medications for hypertension although we reported that non-physicians were able to prescribe medications as a way of task shifting.

Study participants were enrolled as and when diagnosed with hypertension without a control group to compare treatment outcomes. Future research should use more rigorous methods to determine comparative effectiveness and implementation outcomes of the WHO HEARTS-based intervention in diverse populations and settings.

We described the reach and effectiveness dimensions of the RE-AIM framework using mixed methods and adoption, implementation, and maintenance dimensions using qualitative methods alone. Mixed methods would be more informative for all RE-AIM domains.

Our findings present minimal perspectives of how the program could be improved, as most responses are affirmative of the program. This could be a product of our qualitative methodology. Future research should explore opportunities to strengthen the WHO HEARTS-based intervention.

## Data Availability

The datasets used and/or analyzed during the current study are available from the corresponding author upon reasonable request.

## Abbreviations

ART: Antiretroviral therapy
BP: Blood pressure
COREQ: Consolidated Criteria for Reporting Qualitative Research
CVD: Cardiovascular disease
EMR: Electronic medical records
FGD: Focus group discussions
HIV: Human immunodeficiency virus
IDI: In-depth interview
ISS: Immune suppression syndrome
KII: Key informant interview
MJAP: Makerere University Joint AIDS Program
MoH: Ministry of Health
NCDs: Non-communicable disease
PLHIV: Persons Living with HIV
TASO: The AIDS Support Organization
UNCST: Uganda National Council for Science and Technology
WHO: World Health Organization
WHO HEARTS: World Health Organization: Healthy lifestyle counseling; Evidence-based treatment protocols; Access to essential medicines and technology; Risk-based care; Team-based care and Systems for monitoring
